# Preservation of Iodide of Iron

**Published:** 1869-02

**Authors:** 


PRESERVATION OF THE SYRUP OF IODINE OF IRON.—Thomas B. Groves, F. C. S. (*London Pharmaceutical Journal*, March), says: Phosphoric acid is the only acid that can be relied upon for the preservation of the syrup of iodine of iron. He reccommends the addition of one-half fluidounce of dilute phosphoric acid to thirty-one fluidounces syrup iodide of Iron.. The acid should not be added until the syrup is perfectly cool. With this proportion the syrup keeps perfectly well, whether the bottle be full or only partially so.

